# Improving cross-learning in clinical teams using daily on-site reflective meetings

**DOI:** 10.3389/frhs.2025.1630722

**Published:** 2025-09-24

**Authors:** Nawal Khattabi, Ros Axel, Reem AlAbdulmalik, Amal Al-Ali, Erik Hollnagel

**Affiliations:** ^1^ Risk Management and Patient Safety Department, Primary Health Care Corporation, Doha, Qatar; ^2^ Futurum, Jönköping, Region Jönköping County, Sweden; ^3^ Quality and Patient Safety Directorate, Primary Health Care Corporation, Doha, Qatar; ^4^ Australian Institute of Health Innovation, Macquarie University, Sydney, NSW, Australia

**Keywords:** health care teams, learning, safety II, resilient organizations, reflections

## Abstract

**Introduction:**

Learning is fundamental for improving patient safety and quality. Historically, people have focused on learning from unsuccessful performances, such as accidents, incidents, or near-misses. Contemporary approaches to patient safety emphasize the importance of learning from successful everyday work. This approach to learning is less common in the healthcare system and does not carry the same sense of urgency as learning from work that does not go well. Broadening an organization's learning strategies to include learning from everyday work requires adopting new methods and mindsets.

**Methods:**

This study describes the experience of implementing the Resilient Performance Enhancement Toolkit (RPET) in a multisite primary care organization. RPET was introduced through structured daily reflective meetings aimed at fostering cross learning, team adaptation and real-time reflection. Qualitative feedback and thematic observations were collected to explore its impact.

**Results:**

The use of RPET varied across 27 Health Centers (HC), with seven early adopters (29 teams) maintaining consistent practices despite pandemic disruptions. By 2023, meeting frequency stabilized, ranging from daily to monthly. Teams reported improvements in patient safety, communication, and team learning, while identifying challenges such as time constraints and interdepartmental coordination. Key benefits included enhanced teamwork, increased risk identification and improved staff morale.

**Conclusion:**

Embedding reflective practices into daily routines through RPET can strengthen organizational learning and resilience. This approach offers a practical method for shifting healthcare systems toward proactive, Safety-II aligned strategies that support continuous improvement in dynamic clinical environments.

## Introduction

Learning is necessary to improve patient safety and quality in healthcare. Complying with the orthodox safety legacy (1), organizations have focused on learning from unsuccessful performances, such as accidents, incidents, or near-misses (2). However, contemporary safety thinking emphasizes the importance of learning from everyday work that goes well (3).

Healthcare organizations are often characterized as complex adaptive systems (4–6), where risk and safety are emergent rather than resultant outcomes, and where it therefore can be difficult unequivocally to link undesirable outcomes to specific causes. Healthcare system management is therefore gradually changing from a traditional focus on decreasing unwanted events and adverse outcomes to instead increasing opportunities for safe outcomes by enhancing the four systemic potentials for resilient performance (7).

It is now widely accepted that resilient performance requires the potential to respond, the potential to monitor, the potential to learn, and the potential to anticipate (8). This study focuses on how people can learn from everyday work that usually goes well and leads to acceptable outcomes, rather than learning derived only from work that does not go well and leads to unacceptable outcomes (9). Learning is often traditionally indirect and mediated by a team of experts that observe or analyze the work of others and provide delayed feedback—the lessons learned—to the others, usually in terms of what should **not** be done. But learning can also be immediate and allow those who work at the sharp end to learn directly from what they do. This form of learning can be done whilst working with others who share their insights about Work-as-Done—that is how work is actually performed in practice, as opposed to how it is imagined or prescribed. Although this type of cross-learning—defined as informal, or peer-to-peer learning that occurs through shared reflections and everyday attractions, is essential in most work settings, staff shortages, established orthodoxy and unpredictable demands often limit opportunities for team debriefings and reflections (10, 11). Teams rarely discuss what has happened during their working days unless a critical incident has occurred. Although learning from incidents is important, it is limited to what went wrong or did not work (also known as Safety-I) and fails to pay attention to what has gone well and what worked (also known as Safety-II) (12–14). Understanding how systems and processes normally operate not only focuses on what actually happens when work goes well but also provides more robust contextual knowledge that enhances how people make sense of events when they occur. Work that goes well represents “an ongoing condition in which problems are momentarily under control due to compensating changes.” (15).

For many years, it has been acknowledged that organizational learning does not happen in a single step but takes place through several stages (16). However, compared to the interest in organizational learning, relatively little attention has been paid to how people learn directly from their work, how this can change their underlying beliefs and espoused values [(17), p. 118], that determine how they do their work.

**Figure 1:**
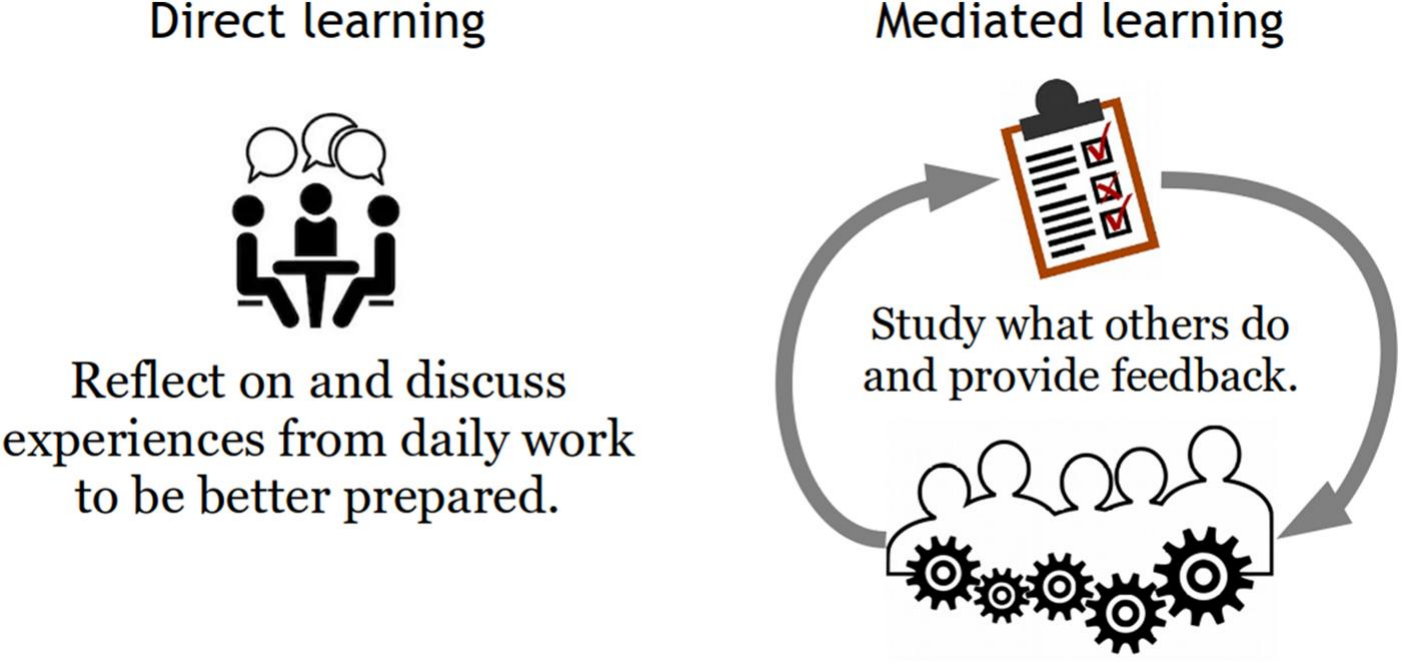
Direct vs. Mediated learning.

Direct learning occurs when individuals can discuss and reflect on their daily work, preferably when they still have good recall of it. By contrast, organizational learning is mediated in the sense that it is brought about by someone else, as shown in Figure 1. Cross-learning among clinical teams should be direct rather than mediated; people should learn directly from the situations they experience, rather than from how others analyze what was reported from their work. Learning should not be limited to what is conventionally reported, but reporting should be driven by the need of learning.

**Figure 2:**
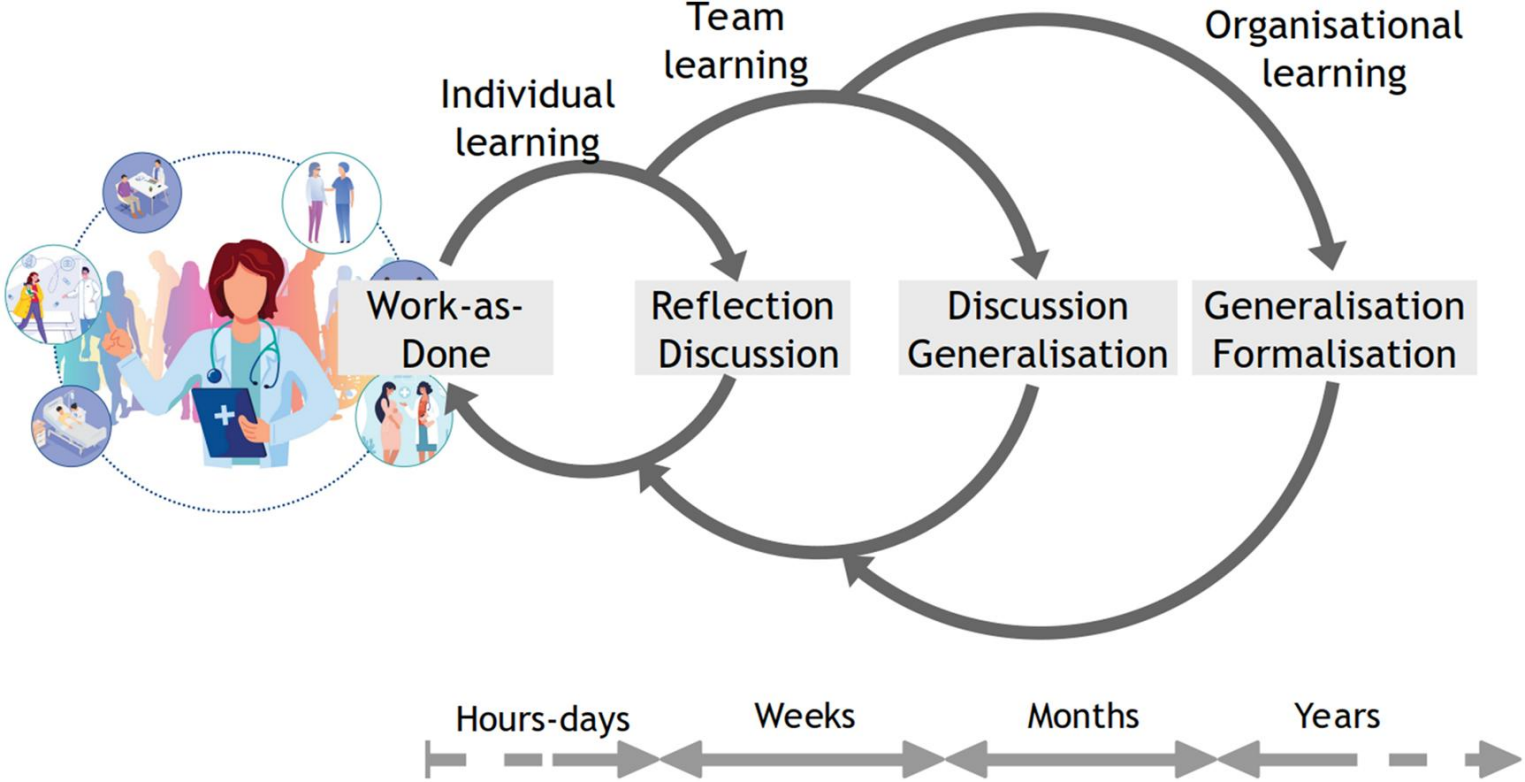
From individual to organizational learning.

Direct learning has two other significant advantages over mediated learning. First, direct learning is immediate, with little or no delay, particularly once it has become a part of daily routines. Therefore, it can be directly applied in the context rather than later through recall or reconstruction. Mediated learning is inevitably delayed, either by weeks, months, or sometimes even years (Figure 2). Second, direct learning is specific and, therefore, relevant to Work-as-Done. Mediated learning is generalized to various degrees because it is interpreted by others and returned as advice or instructions, or even as formal institutionalized procedures (Work-as-Imagined).

The Resilient Performance Enhancement Toolkit (RPET) was developed to enable direct cross-learning among clinical teams by emphasizing daily reflective conversations, and common problem solving (18). RPET, engages teams in collaborative discussions guided by open-ended questions, such as “What went well today?” or “What challenges are we anticipating tomorrow?” This approach aligns with the principles of Safety II by focusing on understanding and reinforcing what went well, rather than just identifying errors, as in Safety-I.

The principle of RPET is to encourage people to consider issues, such as the following:

• What went well today?
• Why did it go well?
• Where there any obvious changes to the routines by you or someone else?
• Did anything surprising or unexpected happen today?
• Did you have to revise or adjust priorities or plans?
• Were there situations that somehow felt different from usual?
• Were there any mismatches between demands (work pressure) and resources?
• Was there anything that, in your view, could have been avoided or done better?

It is essential for RPET, that learning takes place **when** work takes place and preferably as a natural part of work, e.g., at the end of each day. That learning takes place **where** work takes place—from the “coalface” to the boardroom. Learning should be immersed in the work environment and not happen off-site. Learning is **by and for** the people who do the work. Learning should be based on what they know and remember from the work situation, not what they are prompted to discover or reconstruct when others later ask about it.

This study reports the experience of a multi-site primary care system after the introduction of reflective daily learning sessions using RPET. Prior to the program, teams in the healthcare centers (HC) met regularly for projects and for steering the operation of the HCs, but there were no formal or informal meetings to reflect on the work done. Staff break times were often adjusted to ensure the continuity of operations which minimized opportunities for informal conversations among teams.

The aim of the study is both to describe the implementation of RPET across a healthcare system and to describe healthcare staff early experiences of using RPET.

## Method

The program was introduced in a phased approach across 27 HCs to facilitate cross-learning among teams and create opportunities to reflect on work. Initial training was provided to all 27 HCs; however, only 10 HCs proceeded to fully implement the tool as described in Table 1. In the table, an “X” denotes teams that successfully implemented the tool, while shaded cells represent those that did not proceed with implementation.

**Table 1:** Description of where the RPET meetings were implemented across 27 health centers.

Health Center (HC #)	#teams Implemented RPET	Teams Implemented RPET									# Teams in each HC	# Staff in each HC (Dec 2020)	#Patient population visiting each HC	#Patient Visits in 2020 for each HC
		Nursing	Physician	Pharmacy	Laboratory	Radiology	Dental	Physiotherapy	Admin	Home care				
HC A	8	X	X	X	X	X	X	X	X		8	225	43,901	128,479
HC B	7	X	X	X	X	X			X	X	8	157	53,476	89,502
HC C	6	X	X	X	X	X	X				8	263	67,978	146,152
HC D	4	X		X	X	X					8	135	14,807	49,772
HC E	2	X					X				8	349	63,195	238,477
HC F	1	X									8	193	38,596	97,798
HC G	1			X							8	291	126,961	199,501
HC H											8	290	109,668	151,663
HC I											8	52	4,990	11,929
HC J											8	241	69,372	151,732
HC K											8	272	81,675	154,863
HC L											8	208	40,371	98,528
HC M											8	321	121,452	170,894
HC N											8	210	50,845	121,858
HC O	1	X									8	251	56,948	148,737
HC P											8	319	78,503	203,439
HC Q											8	249	65,706	169,717
HC R											8	297	60,266	107,474
HC S											7	171	53,535	93,133
HC T											7	67	3,164	11,536
HC U											6	30	3,352	7,594
HC V											7	41	3,308	11,523
HC W											8	328	43,413	105,653
HC X											8	257	90,493	159,490
HC Y	1	X									8	148	37,737	57,420
HC Z	1	X									8	390	126,356	143,318
HC AA											8	281	40,643	154,827

### Phase I: initial training on RPET

The implementation began in January 2020 with RPET training for team leaders and HCs' patient safety champions across all 27 HCs. The training material was an excerpt from a technical note on “*The Resilient Performance Enhancement* Toolkit” (18). The RPET training sessions focused on the learning process, described as “learning from what goes well” and “how to keep track of the learning process.” The central points focused on where to learn, when to learn, what to learn, and who should learn, as summarized in Table 2.

**Table 2:** Summary characterization of RPET meetings.

WHERE • All levels of the organisation • Happens on-site • Tools are an integral part of existing work environment	WHEN • During working hours • As part of routine work • Timely learning
WHAT • Daily work — all operations • Based on what people know or experience themselves • Not based on what they hear	WHO • Only people who are part of the work • It is for insiders • Not facilitated by learning specialists or other experts

**Figure 3:**
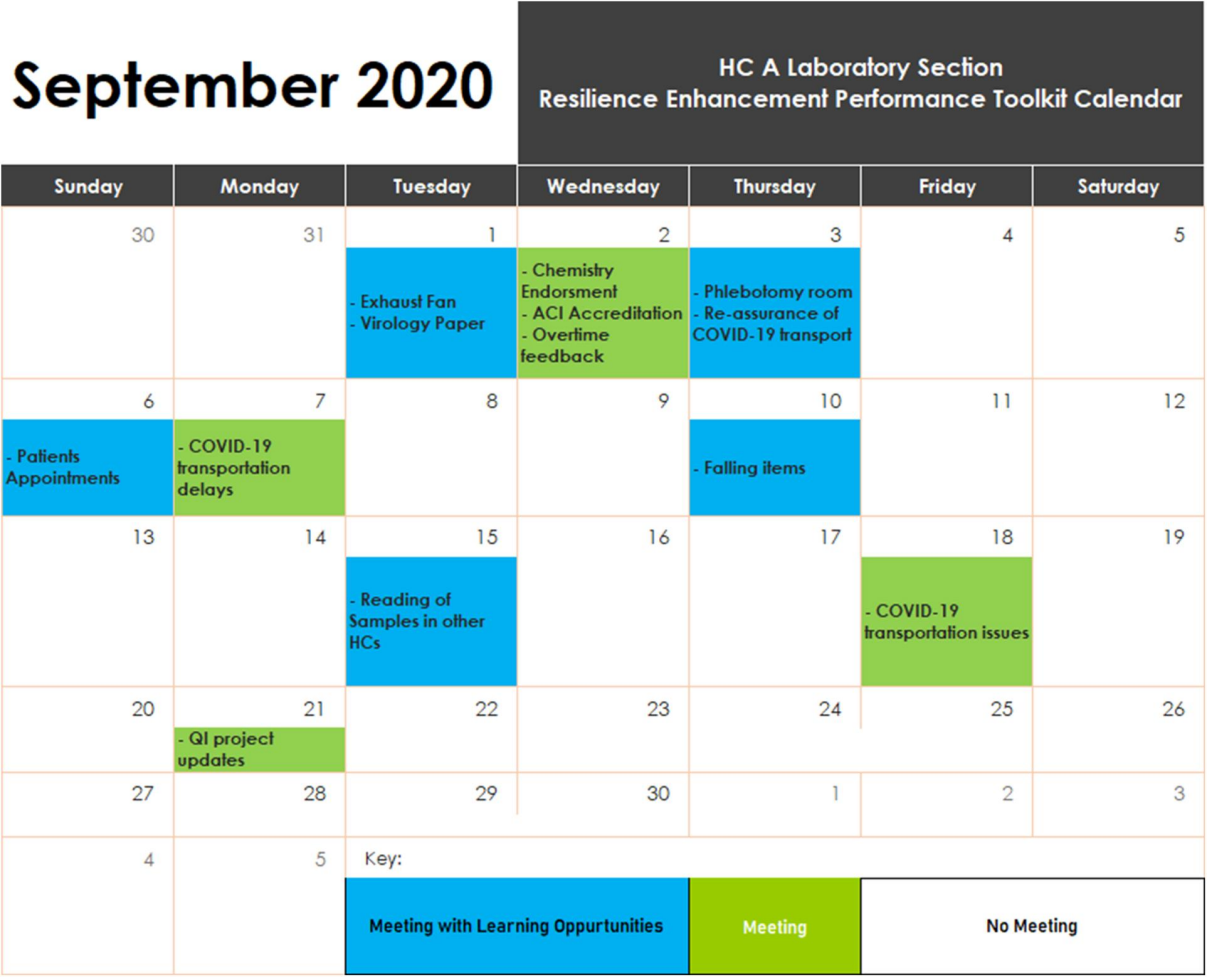
Example of RPET tool implemented in the health center.

The duration of each RPET meeting was planned to not exceed 15 min. The teams were given the freedom to design an RPET calendar to meet their needs; however, all teams agreed upon a common color-coded icon to track meeting days, learning outcomes, and events. Green and white represent days with and without RPET meetings, respectively. The day of the meeting where learning takes place is represented in blue. A sample example of an RPET calendar from one of the HCs is shown in Figure 3 below.

Following the training, the teams were advised to start practicing within their respective professional teams: family medicine and urgent care, dentistry, nursing, pharmacy, physiotherapy, radiology, laboratory, wellness, and reception. Each HC was expected to have seven to nine teams practicing RPET, based on their service provisions, for a total of 211 teams.

### Phase II: training for all clinical staff in October 2020

Phase-II training was conducted during the COVID-19 pandemic, using a virtual platform to accommodate all staff as part of the mandatory annual patient safety awareness session. This concept was introduced to ensure effective safety communications within teams. The training was approved for two continuing professional development (CPD) points for the attending clinical staff. The training consisted of a 40-min lecture, 20 min of questions, and a discussion. The difference between this training and Phase I training is that many of the staff had been participating in RPET meetings prior to Phase II training, which made the discussion more engaging, helped adjust the training material, and clarified key concepts. One of the key points clarified was the difference between an RPET meeting as a reflective meeting and operational meetings, such as monthly departmental meetings (with an agenda focused on the operational plan), huddles (focused on briefings related to daily operational priorities), and patient safety event meetings (focused on a given incident or trend of incidents).

### Phase III: evaluation and consolidation of the RPET practice in December 2020

Towards the end of 2020, the HCs were asked to provide feedback on the RPET sessions. Of the 27 HCs, seven voluntarily shared the RPET calendars they had developed to document learning from RPET meetings, as shown in Table 3. These HCs were considered early adopters based on their proactive engagement and sustained use of RPET. The last author, as an expert and researcher in Resilience Engineering, conducted hourly sessions with each of the seven HCs on different days using a virtual platform. These sessions were structured as open discussions tailored to each team's specific practices and needs, utilizing a structured interview guide. Participants were required to bring their RPET calendars and discuss their experiences using the tool, highlighting any challenges and benefits. Each virtual session involved one HC, with teams selected based on their active engagement with the toolkit, submission of their RPET calendars, and willing to share their experiences. A total of seven sessions were conducted, one session per HC on different days. Altogether, 29 teams participated across all HCs. The HCs shared their experiences using the toolkit and their RPET calendar, along with a short video of one of their RPET sessions they had in their HC. An inductive-deductive thematic analysis was conducted using the six-phase approach outlined by Braun and Clarke (19). Firstly, the authors reviewed the notes and summaries from the evaluation sessions and generated open codes without a pre-defined framework. Examples of initial codes included “difficulty finding time for meetings,” “peer learning across teams,” and “effective communication.” These codes were then grouped into candidate themes such as “time constraints and workload,” “learning opportunities,” and “communication and collaboration.”

**Table 3:** Phase 3 participating teams per health centre.

Health Centers	No. of teams practicing RPET Tool	Teams								
		Nursing	Physician	Pharmacy	Laboratory	Radiology	Dental	Physiotherapy	Reception	Homecare
HC A	8	X	X	X	X	X	X	X	X	
HC B	7	X	X	X	X	X			X	X
HC C	6	X	X	X	X	X	X			
HC D	4	X		X	X	X				
HC E	2	X					X			
HC F	1	X								
HC G	1			X						

Secondly, a deductive lens was applied to align these themes with the four areas of the resilience engineering framework forming the foundation for analyzing the staff experiences in the results section. This mapping allowed for deeper interpretation of how RPET practices reflected key aspects of resilient performance.

The intent of these one-to-one sessions was also to enable the last author to deliver a customized virtual training session tailored specifically to the local progress of the teams in adopting Safety-II and RPET. The Safety-II training was then delivered to all HCs aimed to making the teams' learning stories visible, fostering the benefits and addressing the challenges and misconceptions.

Finally, an evaluation was conducted to determine if the seven HCs, as early adopters of RPET as a Safety-II learning tool, were also HCs that demonstrated the most learning from incidents at the team level. The aim was to identify whether the HCs that learned retrospectively from events were the same HC that also learned from everyday work. To achieve this, data from 2020 risk management reports, particularly the percentage of reported incidents that were reviewed by each HC, were analyzed to identify if there is any relationship between incident learning and learning from everyday work.

### Phase IV: evaluation of sustained RPET practice in 2020–2023

To evaluate and sustain the integration of RPET practices, regular visits to the health centers and interviews with the section leads were conducted. Members of the patient safety team provided support to the HCs' teams to ensure RPET practices were maintained in these health centers and integrated effectively into their schedule.

To promote organizational wide sustainability, RPET was included in the monthly Safety management training as a standalone section for all staff, clinical as well as non-clinical, allowing them to use it as a refresher and ensuring that newly opened health centers and new joiners receive comprehensive training. Additionally, RPET learning materials, including infographic posters and success stories, were continuously shared through emails to all the staff, maintaining engagement and reinforcing learning across all health centers. See Appendix A, for prompt safety-II guide posters aiming to facilitate RPET meetings.

To further encourage the utilization of this initiative, a competition was conducted as part of the 2022 annual patient safety campaign to promote the reflective learning session. The competition encouraged HC staff to showcase their practices for sustained RPET meetings and share key learning experiences.

### Ethics

This study did not require ethical approval as it did not involve human subjects research or sensitive data collection.

### Patient and public involvement

No patients or members of the public were involved in the work.

## Results

The use of the RPET varied among the 27 HCs. Seven of those HCs (consisting of 29 teams) that received training in Phase I consistently used the tool. Notably, despite the disruption caused by the COVID-19 pandemic, which significantly affected work processes, teams from ten HCs continued using the tool without any formal mandate.

A total of 72% of the clinical staff across all the HCs attended the virtual training in Phase II. At the time during the Covid-19 pandemic, many conferences and training programs were canceled, and a few were postponed and moved online, which might have contributed to the high rate of attendance for the RPET training.

**Figure 4:**
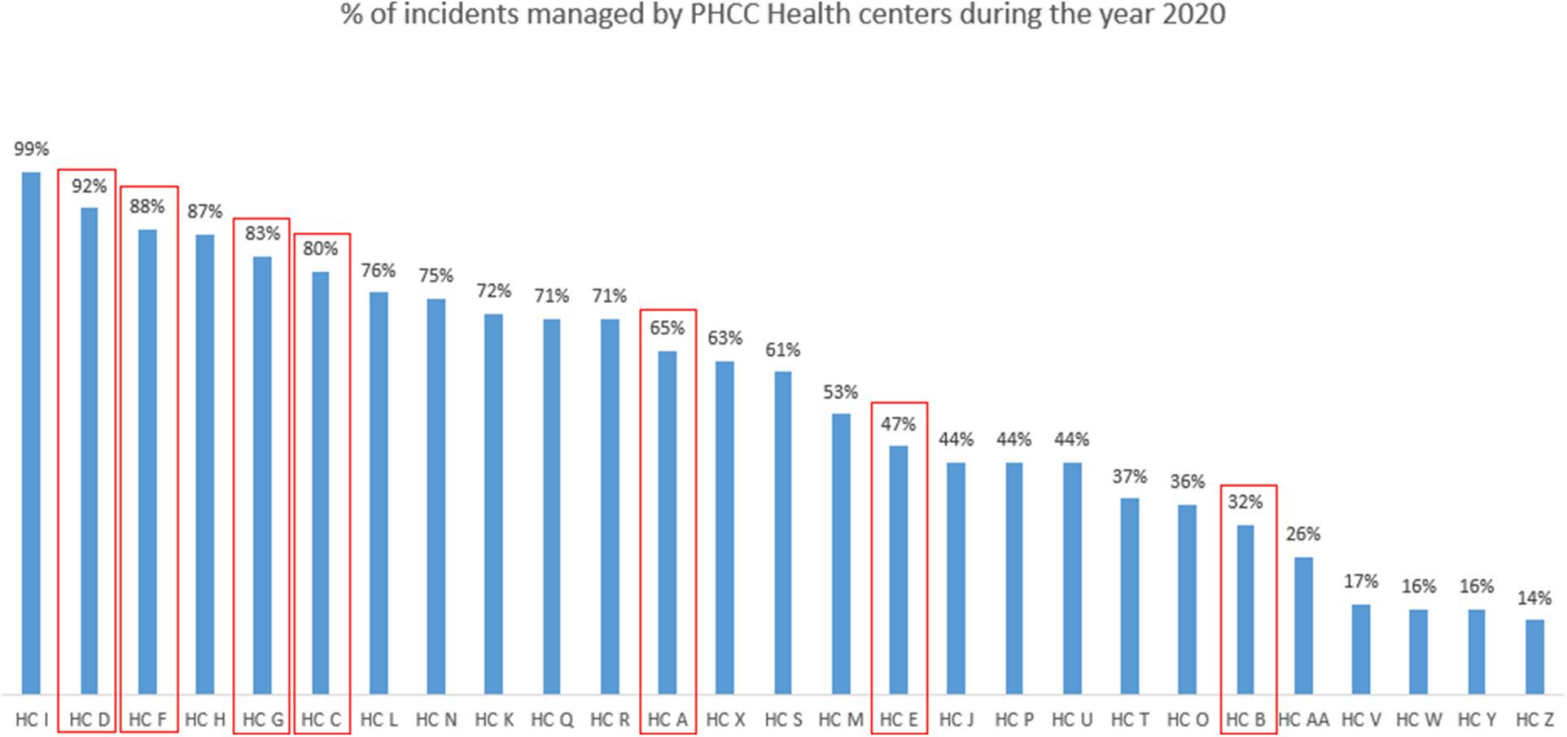
Percentage of incidents managed by PHCC health centers during 2020.

In Phase III, the teams were invited to a one-hour discussion session to share their experiences using the tool. All 29 teams from the seven HCs participated in the consolidation session, shared their experiences, perceived benefits, and challenges. Interestingly, three of these seven health centers had reviewed less than 80% of their reported incidents, yet they demonstrated a strong commitment to learn from everyday work through the consistent use of RPET (Figure 4).

Moreover, the implementation of the RPET tool revealed several benefits and challenges expressed by the staff, which were compiled into a list as shown in Table 4. As these sessions took place during the pandemic, two out of the seven HCs used virtual platforms to share and discuss what was learned. The remaining HCs continued with in-person meetings, while observing infection control measures. The majority of teams, however, considered the program as an unnecessary activity and canceled it during the COVID-19 crisis.

**Table 4:** Benefits and challenges of RPET reported by teams.

Benefits	Challenges
• Opportunity to share individual experience and ideas • Improves interpersonal relationship, teamwork and communication between the leads and team members • Feeling of “being heard” • Sense of pride and responsibility in sharing how situations were managed positively at individual level • Challenges, solutions and opportunities are discussed in the same platform • Promotes work satisfaction • Helps in identifying risks and improvement initiatives	• New concept, new practice and varying perception about the tool • Difficulty in identifying learning from an experience shared by staff • Challenges pertaining to interdepartmental issues • Staff commitment and participation • Documentation in the RPET calendar • Cancellation of gathering as preventive measure to stop the spread of infection

Furthermore, the assessment of RPET meetings utilization across the seven HCs from 2020 to 2023 revealed significant variation in the frequency of meetings between the teams. In 2020, the frequency of RPET meetings ranged from daily sessions in some teams to irregular meetings held as infrequent as once per month. While some teams recorded up to 15–20 meetings per month, others met as little as two to three times a week or only once per month.

By 2021, this variation in frequency persisted, with some teams meeting almost daily while others maintained RPET meetings on a need-based schedule, meeting two to three times per month. The number of times RPET meetings were conducted varied from 256 times a year in one HC to none in others.

In 2022, teams maintained similar patterns, with several teams conducting RPET meetings either once per shift or 20–25 times per month. However, some teams reported conducting RPET meetings three to five times per month or only on an as-needed basis. The maximum recorded frequency for the year was 359 meetings in one team, indicating increased engagement.

By 2023, all teams were consistently maintaining RPET meetings, although the frequency of these meetings continued to vary significantly. Some teams conducted meetings 1–2 times per month, while others held daily sessions or met as frequently as 15–20 times per month. The number of learning meetings conducted ranged from a minimum of 24 times in one team to a maximum of 304 meetings in other teams in 2023. Throughout the four years, some HCs frequently shared feedback through an instant messaging tool at the end of shifts to ensure staff from other shifts could also benefit from the learning and coordination efforts.

**Figure 5:**
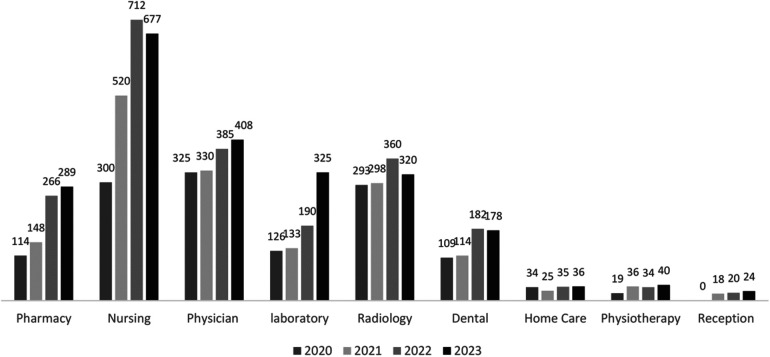
RPET meetings conducted per year by section in seven HCs.

Figure 5 below represents the frequency of RPET meetings held across different teams in the seven HCs from 2020 to 2023. The nursing team consistently reported the highest number of learning meetings while physicians and radiology maintained steady participating throughout the years.

The expert-led training sessions effectively increased the visibility of the team learning stories, the feedback shared by the teams during the consolidation sessions were analyzed using thematic analysis. The thematic analysis is summarized in Table 5. Staff across the seven HCs expressed their experiences through RPET meetings. Using the key themes identified in the analysis, their feedback was classified into four key areas of resilience: responding to challenges, monitoring situations, learning from experiences, and anticipating future issues (20).

**Table 5:** Thematic analysis of feedback gathered during the session.

Theme	Key Observations	Testimonials	Aligned Resilience Area
Patient Safety Improvement	Feedback emphasized improved patient safety and outcomes	– “Improve day-to-day risk and opportunities related to patient safety”. – “Everyone is contributing to explore factors behind patient safety incidents”	Responding/Monitoring
Learning Opportunities	Positive learning opportunities and educational value were noted	– “Through RPET meetings, we discuss how can we improve the care and have less mistakes.” – “Great chance to learn how the team overcomes the daily challenges and learn from each other.”-	Learning/Anticipating
Communication and Collaboration	Communication and collaboration were highlighted as effective in fostering teamwork	– “Effective way of mutual communication and discussing how our work went well through the day.” – “Feedback from staff can help in some work arrangement”	Responding/Monitoring
Time Constraints and Workload	Staff frequently mentioned time constraints and workload as barriers to practicing RPET.	– “We struggle sometimes to secure a time where all can meet due to busy clinics.” – “Most of the time, we practice RPET through Whatsapp or Microsoft teams”	Responding/Anticipating
Challenges in Implementation	Challenges included staff shortages and resources limitations	– “Useful but can't be implemented easily in our setting.” – “Staff shortage and busy clinics made it difficult to conduct regular RPET meetings”	Anticipating

### Potential to respond

Several staff members highlighted how RPET helped them adapt to unexpected situations, allowing for rapid adjustments in their daily work. For example, radiology staff from one HC shared that their participation in RPET enhanced their ability to identify and mitigate risks related to equipment security.

“RPET meetings made it easier to identify risks with the x-ray lock code. We realized that the code could be compromised, so we worked with the vendor to change it immediately, ensuring patient safety” (Radiology team, HC B). This aligns with the thematic analysis theme of Safety Improvement, emphasizing how teams responded effectively to immediate challenges.

Another notable example from the nursing team emphasized how RPET meetings facilitated collaboration and addressed issues related to oxygen cylinders that were not properly stored.

“RPET meetings helped us identify that empty oxygen cylinders were scattered in different areas and not secured with a chain. We have identified a room in the basement to store them properly and they are now secured with a chain” (Nursing team, HC B). This further illustrates the theme of Collaboration in addressing safety concerns.

### Potential to monitor

Multiple examples of monitoring were identified during the RPET discussions. In a dental clinic, for example, the team described how they used RPET to monitor and mitigate risks related to supplies shortages.

“Following a RPET meeting, we started now to monitor our supplies every two weeks to avoid shortage and the stop of services that will affect the patient care” (Dental team, HC E). This reflects the theme of Communication and Collaboration, where regular RPET meetings enabled effective tracking of critical resources.

In another instance, the nursing team noticed that the IP phone code-blue broadcasting system was not always functioning correctly, which posed a safety risk in case of emergency communications.

“During one of the RPET meetings, we highlighted the necessity of monitoring the IP phone code-blue broadcasting system, so we now perform a broadcast test every morning at the beginning of the shift to ensure the system is working properly across the HC” (Nursing team, HC B). This example ties to the theme of Safety Improvement, demonstrating how monitoring processes were established to ensure reliability.

### Potential to learn

Learning was the most frequently mentioned concept across all HCs. Staff indicated that RPET allowed them to reflect on both positive and negative experiences, resulting in improvements in their daily practices. Pharmacy team, for instance, explained that RPET discussions led to actionable changes that optimized medication distribution processes.

“Modifications to our workflow following a RPET discussion made it easier to distribute drugs and ensure the patients receives their right medications” (Pharmacy team, HC D). This is connected to the thematic analysis theme of Learning Opportunities, as team adapted their processes based on lessons learned.

Another significant learning experience came from the laboratory team, where an issue with the oral glucose tolerance tests (OGTT) solutions was identified. The unpleasant taste caused reluctance among pregnant women to take the solution during testing, which affected patient care.

“RPET meetings helped us realize that the bad taste of the OGTT glucose solution was a recurring issue. One time, a patient brought in her own solution, but it was cold. We used this as an opportunity to discuss the issue with the team and learn from it. Now, we provide the OGTT solution cold to patients” (Laboratory team, HC G). This also illustrates Learning Opportunities, showcasing how reflection during meetings led to practical challenges.

### Potential to anticipate

The pharmacy staff discussed how RPET allowed them to anticipate and address issues related to high-alert medication handling.

“We discussed potential risks with high-alert medication errors during RPET meeting and implemented preventive measures to avoid them like; shelves separation” (Pharmacy team, HC G). This aligns with the thematic analysis theme of Challenges in Implementation, as teams anticipated future risks and adjusted accordingly.

Another example from Radiology showed that some protocols were changed based on learning shared in RPET meeting.

“A technician shared that a wrist x-ray (posteroanterior and lateral views) was initially ordered by the doctor for a patient with a wrist injury, both views showed normal. However, the technician decided to add an oblique view, which revealed a hairline fracture, and since that day as a learning, it has been decided to perform three views—posterior, lateral, and oblique—for all wrist injury cases. A week later, this change proved effective as a subsequent wrist injury case was diagnosed with a fracture visible only in the oblique view.” (Radiology team, HC C). This reflects the theme of Learning Opportunities, as forward-thinking strategies emerged from the meetings.

In phase IV, the sustainability of RPET practices was achieved across all the seven health centers with consistent implementation observed from 2020 to 2023. Regular RPET meetings were maintained, and at least one team in each health center effectively incorporated the practices into their daily schedule.

The 2022 patient safety campaign competition, which received 20 submissions from different teams across all HCs, demonstrated significant engagement that highlighted sustained practices of RPET across various teams. Among these submissions, nine were from the teams of the seven early adopters HCs, while the remaining 11 submissions were from teams of the nine HCs that adopted the tool later (Table 6). The submissions represented a variety of expertise, including Physicians, Nursing, Pharmacy, Laboratory, Radiology, Dental and Wellness, highlighting the tool diverse application across the teams. This competition not only recognized the efforts of the existing successes but also inspired others to adopt and sustain RPET meetings across the organization.

**Table 6:** HCs and teams participating in the 2022 annual patient safety campaign-RPET competition, including early adopters highlighted in grey.

HC	Team
HC C	Physician
	Nursing
HC J	Nursing
	Laboratory
	Radiology
HC A	Dental
	Pharmacy
	Nursing
HC F	Nursing
	Laboratory
HC AA	Pharmacy
HC Q	Nursing
HC G	Pharmacy
HC M	Nursing
HC O	Pharmacy
HC E	Radiology
HC K	Radiology
HC X	Nursing
HC H	Nursing
HC Z	Wellness

## Discussion

Reaching safe outcomes for patients does not always happen in the same way or via expected paths but can sometimes be the result of daily and habitual adjustments (7). In this study, RPET meetings enabled teams to appreciate the value of sharing their experiences of the challenges they faced and how they routinely overcome these challenges. Learning moved gradually from being triggered by outcomes (incidents and near-misses) to being prompted by the overall resources and processes available that day, environmental challenges, and changes in patient influx. This is evident from the commitment demonstrated by the HCs, as noted in the results section, where even those that had not reviewed all their reported incidents consistently used RPET to address and learn from how everyday work challenges and opportunities were managed. This indicates that teams are committed to daily reflective meetings are not necessarily those who show consistent learning from incidents. Another key finding is the sustained increase in using RPET over time. This sustainability was likely driven by tailored training, one-to-one meeting sessions, and continuous feedback. Ros et al. highlighted that sustaining improvement in complex systems requires continuous engagement and the ability to adapt interventions to evolving needs (21). Consequently, this type of sustained engagement through RPET makes the learning approach particularly appealing to clinical teams, as it improves the staff's ability to notice and communicate what was happening in the meetings, thereby potentially improving the team's ability to monitor and anticipate challenges. However, Wahl et al. noted that although safety huddles can support system resilience through learning and responding, reflecting on positive events was often more challenging for staff compared to addressing adverse events. Improving processes and competencies will support the teams' abilities to respond to similar situations in future (22). RPET meetings facilitate learning by reflecting on how services are delivered despite challenges, aligning with Safety-II principles. As shown in Appendix A, Safety-II type questions prompt teams to reflect on successful adaptation, fostering a proactive learning environment and overcome challenges.

Learning is focused on “how” services are delivered despite challenges and has been shown to increase learning and responding capabilities in HCs conducting RPET meetings regularly. This is further supported by the patient safety culture survey results, which identified organizational learning and continuous improvement as key strengths, which reinforce the role of RPET meetings in sustaining a culture of continuous improvements (23). It was also reported that the teams felt that the individual experiences shared during reflection meetings provided collective learning. Peer learning, important for improving competency, was supported by the findings of Putri and Sumartini (24), who emphasized the value of shared experiences in clinical education.

While the initial intent of implementing RPET meetings was to provide opportunities for cross-learning among teams, the generated learning was found to improve four capabilities of resilient performance: monitoring, anticipating, responding, and learning. In addition to the cross-learning that resulted from the RPET meetings, the teams shared that they experienced more team engagement and cohesiveness. When urgent care nurses learned more about what triage nurses do daily, they gained more insight into why certain things happened in certain ways and avoided making assumptions or misinterpretations. When triage nurses learned more about patients' care and management post-triage, they understood how triaging patients is not only based on the triage policy (Work-as-Imagined) but also on the dynamic reality of that specific day (Work-as-Done). This communication in RPET meetings improved the understanding of the context, consequently reducing the tension between staff working in related clinical teams.

The results show that RPET meetings give teams opportunities to come together and share what otherwise usually goes unnoticed, namely, what works well. Initially, the staff were not sure how to talk about work when no incident occurred. The difficulty in detecting and noticing what goes well is acknowledged as a “real and serious problem.” (18, 22). Identifying 'safe adaptations' that result from the “fluency” staff gain in undertaking their tasks under varying difficulties, requires facilitated activities (25). When the teams started using Safety-II type questions (Appendix A), they realized that there were opportunities to learn from how challenges were overcome. Identifying Safety-II type question to facilitate Safety-II practices was demonstrated in other studies like Provan et al. (26) and Bentley et al. (27).

The study found that reflective daily learning sessions can significantly improve collective and cross-learning among clinical teams. By focusing on daily reflections, teams can identify opportunities for learning, uncover challenges, strengthen local adaptations, and improve communication. Face-to-face meetings helped to build trust among teams, impacting on information sharing and learning (15). The results of this study showed that providing different teams with the opportunity to meet daily for as little as 15 min has been shown to increase teams' understanding of environmental factors, organizational structures and dynamics, patients, communities, safety events, and employees' anticipation.

Organizations need to encourage local reflection practices as a key feature of learning, as mentioned by Edgar Schein: “a learning culture must value reflection and experimentation and must give its members the time and resources to do it” [(17), p. 119].

## Limitations

This study has several limitations that should be considered when interpreting the findings. First, the study was conducted within a single organization that is multi-sited in Qatar, which may limit the generalizability of results to other healthcare systems or cultural settings. Second, RPET adoption varied across HCs, with only a subset of HCs demonstrating sustained implementation during the study period. Thirdly, the data were primarily self-reported through facilitated discussions, which may introduce bias or overrepresentation of positive experiences. Lastly, the study did not include a comparator, limiting the ability to determine whether the observed outcomes were directly attributed to the RPET intervention. Despite these limitations, the insights generated offer valuable evidence on the feasibility and perceived value of embedding Safety-II principles through daily reflective practices in primary care settings.

## Implications

This study demonstrated that it is feasible for healthcare staff to learn continuously from everyday work. Reflective meetings show how performance can be improved directly through experience at both the individual and team levels. This method eliminated learning delays.

The results of this study encourage further applications and practices of learning from everyday work, potentially transforming safety learning into encompassing learning from what has gone well in addition to learning from safety events.

## Figures and Tables

**Figure 6:**
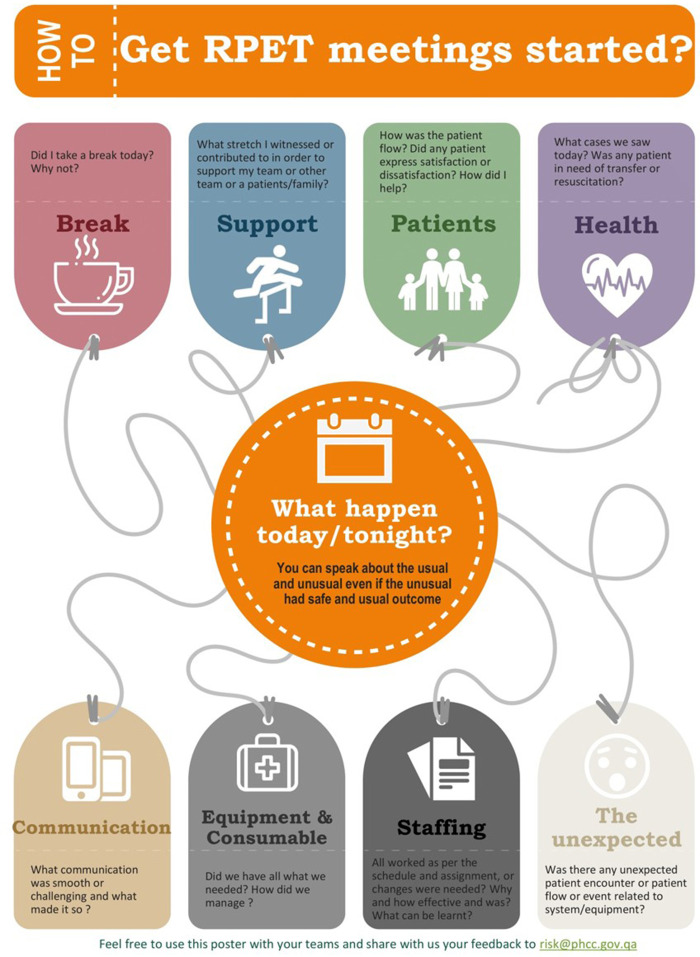
Safety-II type questions to prompt discussion and facilitate reflective safety-related meetings in the health center.

**Figure 7:**
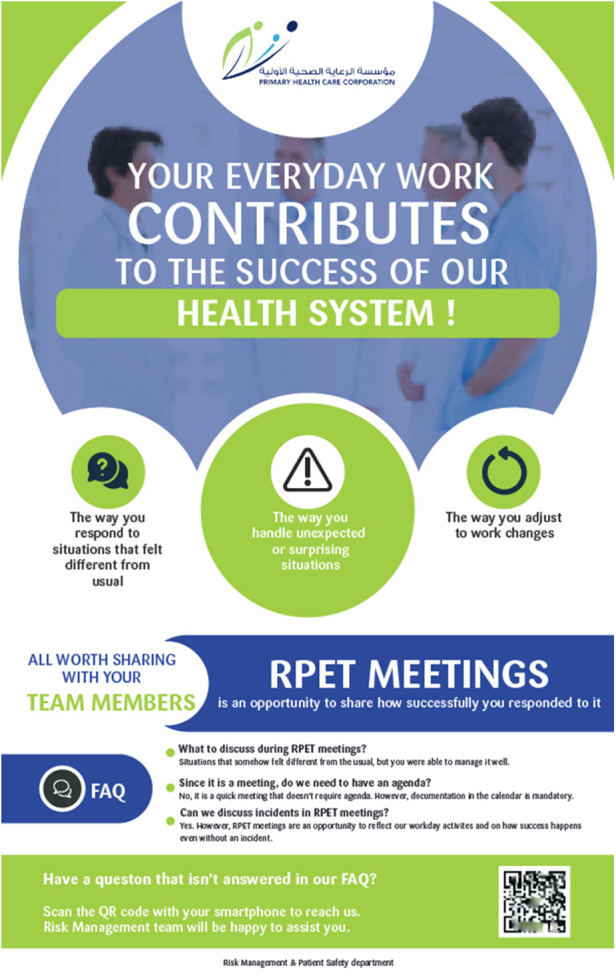
Supporting tools distributed to staff.

## Data Availability

The original contributions presented in the study are included in the article/Supplementary Material, further inquiries can be directed to the corresponding author.

## Appendix A

This appendix includes infographic posters of prompt safety-II type questions to facilitate RPET meetings in the HCs.
