# Supplementary material for: Improving cross-learning in clinical teams using daily on-site reflective meetings
Source: Front Health Serv. 2025 Sep 24;5:1630722. doi: 10.3389/frhs.2025.1630722 (PMC12504320; doi:10.3389/frhs.2025.1630722)
Supplement: Supplementary file 1 [file Datasheet1.pdf]

RPET

The Resilient Performance Enhancement Toolkit[1]

How to learn from work that goes well and how to use it to do better

© Erik Hollnagel, 2019

Introduction

The traditional focus of safety is on what happens when something goes wrong, when the outcomes of work differ from what were intended and expected. Safety, or rather Safety-I (Hollnagel, 2014), is defined as the absence of harm and injury and the purpose of safety management is to prevent accidents and incidents – large and small – from happening. Safety-I is about not having accidents and the challenge is to find out about their causes in order to eliminate or encapsulate them. Conventional wisdom also argues that there is more to be learnt from accidents than from incidents, more to be learnt from major accidents than from minor ones, and so on. This shows itself in practice as a clear relationship between the severity of an event (magnitude of losses, number of injured and dead) and the time and efforts that are invested to understand what happened and to learn the right lessons.

The principles and practices of Safety-I are governed by the "logic" of the *causality credo*, which can be stated as follows: (1) adverse outcomes happen because something has gone wrong; (2) if enough evidence is collected it will be possible to find the causes and then eliminate, encapsulate, or otherwise neutralise them; (3) since all adverse outcomes have causes, and since all causes can be found and dealt with, it follows that all accidents can be prevented, i.e., the Zero Accident Vision. The rationale for the causality credo has been expressed as follows:

"Sound business procedure, as in fact sound common-sense procedure with regard to the arts or sciences in general, substantiates the thought that the cure of a given

1 RPET is pronounced /rɪˈpiːt/ and is therefore a homonym of 'repeat'.

troublesome condition depends primarily upon knowledge of its cause and the elimination, or at least the mitigation, of that cause. That this principle applies to the prevention of industrial accidents cannot be denied. Success depends also upon the will to achieve and, later, upon ability to apply a known remedy" (Heinrich, 1931, p. 38).

It is, of course, natural to try to be free from harm and injury, whether as a person or an organisation. Accidents and incidents are unwanted occurrences and it makes good sense to try to avoid them as far as possible. Yet in the rush to learn from what has gone wrong two important facts are missed. The first is that most of what happens, indeed nearly everything that happens, usually goes well. It would therefore seem reasonable also to try to learn something from that. Learning from failures alone is not only marginal, it is also expensive and mostly ineffective. The second fact is that if there are causes for what goes wrong then there must also be causes for what goes well. From a Safety-I perspective the two types of causes must obviously be different; otherwise eliminating the causes of accidents would also reduce the likelihood for work to go well.

Resilience Engineering and Safety-II argue that an organisation should learn from everything that happens, from failures, from successes, and from everything in between. Adverse outcomes do not happen because something fails but because system adjustments are insufficient or inappropriate. Work that goes well is not the result of the effective elimination of hazards and risks but rather represents "an ongoing condition in which problems are momentarily under control due to compensating changes [in components]" (Weick, 1987). Safety is therefore a condition where as much as possible goes well and where consequently "nothing" happens. The coveted state of freedom from harm and injury can be achieved by focusing on the so-called "non-events", by making sure that everything functions well and making sure that the "non-events" happen, but not by focusing on the events and by preventing that something fails.

Events and "non-events"

The practical problem is, of course, how this should be done. One obstacle is the psychological phenomenon called *habituation*. This means that we stop noticing something if it is always there and if it happens all the time. We quickly become so used to the adjustments and workarounds that are part of everyday work – and indeed everyday life -

© Erik Hollnagel, 2019

that we do not consider them worth mentioning. Work not only goes well all the time but we also expect it to do so. When it happens it is therefore not surprising, and we therefore gradually stop paying attention to it. The conundrum is that reliable outcomes are constant, which means that they do not attract attention. But in order to improve performance we must find ways to pay attention to them and learn from seemingly trivial details.

Terminological asymmetry

A second obstacle is the lack of readily available terminology, categories and methods. For accidents and incidents we have a well-developed terminology to describe them and their outcomes, many methods to analyse them, and a number of models to explain their purported causes. This makes it easy to notice them, to describe them, to document them, and to share that information with others. But there is no similar terminology to characterise work that goes well, let alone methods to analyse it or models to explain and understand it. Indeed, in common parlance an absence of accidents means that "nothing" happens. And if "nothing" happens then there is clearly nothing that can be observed and nothing that can be learnt. This is, however, essentially a chicken or egg dilemma, since once we find ways to describe what happens every day, to perceive what cannot be seen, it becomes obvious that there is much of value to learn.

Work-as-Imagined and Work-as-Done

Planning what to do rests on assumptions about how regular the work context is, what the demands and resources will be, how reliably others will perform, and so on. Trying to anticipate what may happen is, however, not like playing a game of chess. No actual situations are as orderly and constrained as a board game, and the 'opposition' in real life rarely behaves as imagined but seems either not to follow the rules or to follow different rules. The planned work – Work-as-Imagined – will therefore never correspond precisely to the actual work – Work-as-Done – no matter how meticulous the planning is. In order to do their work, people and organisations must adjust what they do to match the conditions – unless, of course, they are powerful enough to adjust the conditions to match their plans. The adjustments will furthermore be approximate rather than precise for the very reasons that make them necessary in the first place.

© Erik Hollnagel, 2019

It is important to point out that the issue is not whether WAD is ‘ right’ and WAI is ‘wrong’, or vice versa. WAI and WAD are simply and irreconcilably different. People manage to do their work despite rather than because of all the instructions, policies, procedures and rules that they have been given by well-intentioned policy makers, system designers, and managers. It is essential that organisations try to learn from that rather than pass it in silence.

Resilience engineering and Safety-II have convincingly argued that performance variability and performance adjustments on the whole are strengths rather than liabilities, and that they are the primary reason why socio-technical systems function as well as they do. Humans are extremely adept at finding effective ways to overcome problems at work, and this capability is crucial for safety and productivity throughout an organisation. It therefore stands to reason that there are valuable insights to be gained from looking at Work-as-Done, which means looking at and learning from work that goes well. An added bonus is that it in the long run may strengthen a culture of inquiry and wisdom and gradually weaken the conditioned tendency to focus on the negative.

Continuous Learning

Learning based on accidents, learning from what has gone wrong, is not continuous for the simple reason that accidents are rare and furthermore are supposed to be so. (If they were frequent, an organisation would be unlikely to survive.) Accidents are *stochastic* in the sense that it is uncertain when the next one will happen. When they do happen, there is an understandable rush due to the fact that we do not know when the next event will occur, hence a need to be (reasonably) finished with extracting the lessons from the previous one. This poses a problem for the organisational support from learning – which in practice usually becomes support for reporting and analysis, rather than for learning.

Learning is most effective if it is ***continuous*** and not tied to infrequent or unusual situations, as the case is in Safety-I. Learning should be an integral part of work and as such be given the necessary time and resources. Learning should take place regularly – if not every day then at least every week. Learning from what goes well is part of the thoroughness of the present that is necessary for the efficiency of the future. Indeed, learning from what goes well need not wait for an “event” to happen, because work goes well all the time.

© Erik Hollnagel, 2019

Learning from work that goes well

Learning from what goes well is deceptively simple. All it takes is that we look at what happens each day, try to understand why it goes well, and try to learn from that. This has a number of practical implications.

- Learning should take place ***when*** work takes place and preferably be a part of work. If that is not possible, then it should take place as soon as possible. If learning is delayed people may be unable to recall trivial but important details of their work and of what happened when "nothing" happened. This may to some extent be compensated by ensuring that learning opportunities are regular and seen as a natural part of what goes on in the workplace.
- Learning should take place ***where*** work takes place, on all levels of an organisation from the "coalface" to the boardroom. Learning should be immersed in the daily working environment and not happen off-site. If learning requires "tools", these should be an integral part of the existing work environment. Ideally, as learning becomes part of the daily routine, it should require no extraneous "tools" at all.
- Learning should be by and for the people who are ***part of*** the work. Learning should be based on what people know and remember from the work situation, not what they discover by asking others about it. Learning should not be the prerogative of specialists, such as a learning team or a Human Resources Department. Learning should be by and for insiders and rather than by and for outsiders and should not require skills that are not already possessed by the learners.

Although learning from what goes well really ***is*** simple, it may at first look as a formidable problem because it is unfamiliar to most people. It is therefore helpful to describe how it can be done in a little more detail and illustrate that with an example. The two central issues are the learning process itself in the sense of knowing what to look for and how to manage or keep track of the learning progress.

The learning process: Knowing what to look for

The "secret" in learning from work that goes well is to know what to look for. It is true, of course, that nothing unusual or spectacular happens during regular everyday performance, hence that nothing automatically attracts attention. Yet in reality an amazing number of things happen, even though they go unnoticed at the time. This is mainly due to the

© Erik Hollnagel, 2019

terminological asymmetry described above. The default assumptions seem to be that work goes well because systems are well designed, well built and scrupulously maintained, because designers and managers have foreseen and anticipated everything that could happen, because procedures are correct, complete, and always up-to-date, and because people behave as they are expected to – as they are taught.

A steadily growing number of studies have, however, shown that these assumptions are incorrect and that work mainly goes well because people adjust what they do to match the conditions – including what others do. A starting point for discussing how work goes well is therefore to look for how this is done. Most adjustments are fortunately highly regular and fit into a few types or categories. One category describes how people recognise changes to the working conditions. Another how people handle unexpected situations. And a third category how they come to recognise patterns and contexts. Each category should be addressed during discussions, although not necessarily in any fixed order. To help with that, the following groups of topics illustrate what such discussions could be about. The topics listed here may serve as a starting point, but are neither compulsory nor exhaustive.

How people recognise changes to conditions / situation

Recognising or realising that a situation somehow has changed, that it is different from what was expected, is the prerequisite for responding. This can be the subject for discussions using issues such as:

- Situations where something surprising or unexpected happened
- Mismatches between demands (work pressure) and resources.
- Obvious variability or change in routines, either by yourself or by others.
- Situations that somehow felt different from the usual.
- Situations where the preparations / plans had to be revised or adjusted.

More generally, try to give examples of unexpected conditions such as: interruptions / changing external or environmental conditions / reduced time / ineffective or defective tools or equipment / incomplete, incorrect or surprising information / problems (delays) with communication / lack of documentation?

© Erik Hollnagel, 2019

How people handle unexpected situations

Work can usually be carried out as planned when it begins, although it always will be necessary to make smaller – or sometimes – larger adjustments on the way. It is important to pay attention to them, since they are the main reason who things usually go well. This can be discussed by considering the following issues:

- Situations where it was necessary to make goal trade-offs or change priorities.
- Situations that required a change to the prepared order of actions or operations.
- Examples where work was delegated to others or where others lent a helping hand.
- Situations where something had to be delayed or postponed.
- Examples of shortcuts or alternative ways of doing something.

How people recognise patterns over time

With experience it becomes possible, or even inevitable, to see patterns in the daily work situations. Recognising such patterns can be of great value in preparing for work and in responding quickly and efficiently when something happens. It is therefore useful also to discuss the patterns that are recognised and how they have been discovered. Such discussions can provide a forum for staff to discuss their work and develop a shared understanding of how goals are set and progress made. This does not have to be done every day or discussed as often as the other topics, but perhaps on a monthly rather than a daily or weekly basis. The following issues may help to focus the discussions:

- Give examples of recurrent situations that they have become part of the daily or weekly routines.
- How are recurrent situations used in the preparation for work – including training?
- Is there a reasonable balance between routine and non-routine situations in your work?
- How is experience with recurrent situations captured, analysed, and used for learning?
- Do you informally discuss your experiences from recurrent situations with colleagues?

Learning – from discovery to recognition

Until learning from what goes well has become a routine practice, the discussions will often be opportunities for discovery. By looking at what goes well and trying to understand how it happens, a number of regular features of everyday work will be discovered. But as time

© Erik Hollnagel, 2019

goes by, patterns begin to emerge and the discussions will more often rely on recognition than on discovery.

The purpose of discussing work that goes well, as well as the more institutionalised analysis of reportable events, is not just to understand how adjustments to work commonly are made but also to think about what happens when the same adjustments are made under different conditions or when multiple adjustments come together for one reason or the other. Understanding how work is done and why it is done in a certain way is an indispensable precondition for thinking about how to change it.

Keeping track of the learning progress

Learning should be part of the daily work and take place in close connection to it. This is rarely a problem in the case of accidents and incidents where established practices demand an immediate response. But there is no similar tradition for learning from work that goes well. One reason is that the outcomes are inconspicuous, another that they happen regularly and continuously. There is therefore a need to provide some kind of tool that can help to keep track of and support this activity. A current prototype, developed by Zerprize Ltd. (www.zerprize.co.nz), looks like this.

Icons for different types of days

It is first of all necessary to define some icons that show whether the events of a day have been discussed with the aim to learn something and what the outcomes were. Figure 1 shows how this can be done in the prototype RPET tool, where the colour coding has the following meaning (the coding has arbitrarily been assigned to the dates from 02/04 to 02/12):

- A day gone, but not yet discussed (grey) – 02/04
- A red safety related event – 02/05
- An amber safety related event – 02/06
- A yellow safety related event – 02/07
- A day discussed (green) – 02/08
- A lesson learnt (red with green border) – 02/09
- A lesson learnt (amber with green border) – 02/10
- A lesson learnt (yellow with green border) – 02/11

© *Erik Hollnagel, 2019*

- A day coming, an upcoming or future day that has not yet occurred (white) – 02/12

© Erik Hollnagel, 2019

© *Erik Hollnagel, 2019*

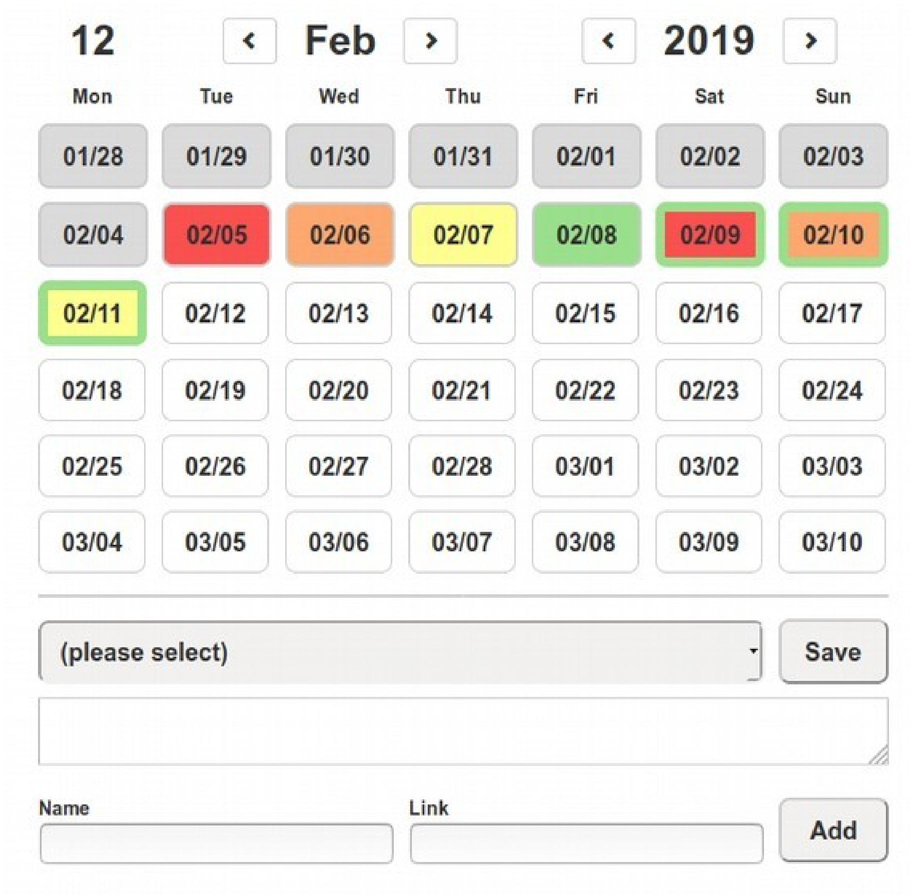

Figure 1

Representation of progress

To support continuous learning it is necessary to keep track of the progress. The obvious choice would seem to be some kind of timeline. While this might be considered for work projects that have a defined length, it is not really practical for continuous work such as at a building site, in a hospital, or in a factory. Here it would be better to group days into chunks depending on the nature of the work and the local culture. (If we as an example consider a hospital – or an airline – that functions 24/7, work days can conveniently be grouped into chunks of seven.)

The solution chosen by the RPET tool (Figure 1), is a continuous calendar where past or future dates can be used by choosing the appropriate month and year. For each day the status can be marked using the colour coding described above. The discussions can be summarised in the scrollable text box below. If additional documentation is available their

© *Erik Hollnagel, 2019*

name and location (***link***) can also be provided. Clicking on the ***save*** button will obviously save all that has been entered.

An example

During the last months of 2018 and continuing into 2019 the RPET was tried at a hospital in Sweden. The RPET tool was not available at that time, so the users relied on a paper version. Their experiences are currently being discussed and evaluated.

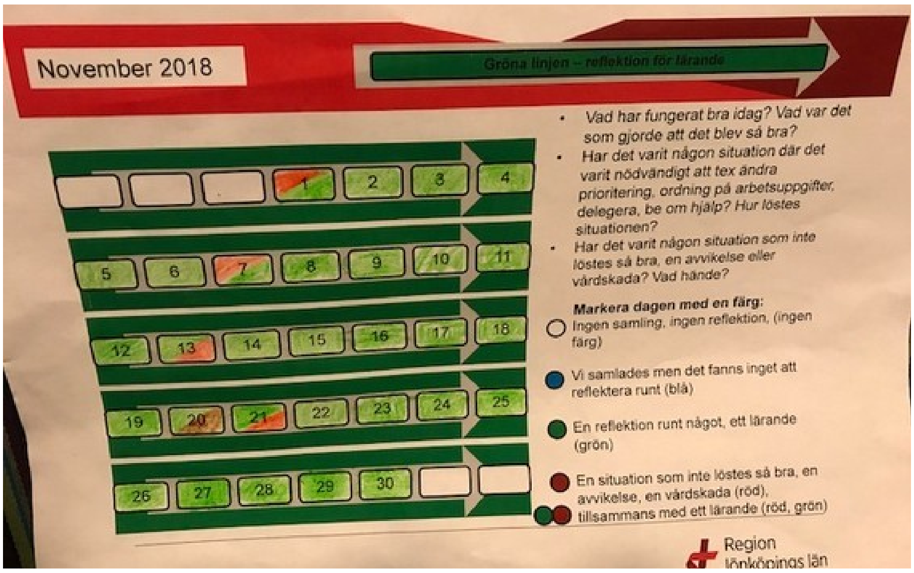

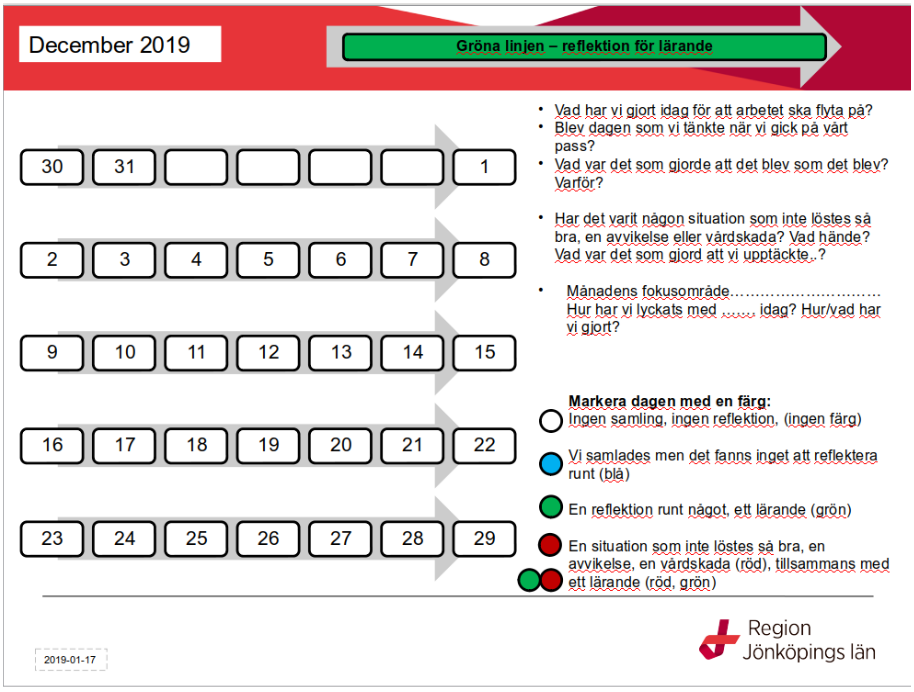

Conclusions

The need to pay attention to and learn from everyday performance is beginning to be widely recognised. This Technical Note summarises the rationale for the need and also outlines a way of how an organisation can begin to learn from everyday practice. Once the process has been started, it will undoubtedly lead to other ideas and practices. This is only to be encouraged since each organisation will have specific needs and potentials that must be addressed.

It is essential to remember that the primary purpose of the discussions is to provide the people at the workplace with an opportunity to learn from what they do; the purpose is not to collect data for others to look at, although it may be an added benefit. The RPET should be used by the sharp end – the operational level – and also by the blunt end – the management level – so that each can learn from their everyday experiences. It should definitely not be used by the blunt end to learn about the sharp end. It is also essential to keep in mind that the discussions never must become a way of questioning people or of scrutinizing their work. There is little to learn from what people may have done wrong – which usually means what others think they have done wrong. But there is a lot to learn from what they have done well, even though they may have become so used to it that it is no longer noticed.

References

Heinrich, H. W. (1931). *Industrial Accident Prevention* (First Edition). New York: McGraw-Hill Book Company.

Hollnagel, E. (2014). *Safety-I and Safety-II: The Past and Future of Safety Management*. Farnham, UK: Ashgate.

Weick, K. E. (1987). Organizational culture as a source of high reliability. *California Management Review*, *29*(2), 112-128.

© Erik Hollnagel, 2019
